# Fractional model for MHD flow of Casson fluid with cadmium telluride nanoparticles using the generalized Fourier’s law

**DOI:** 10.1038/s41598-021-95528-z

**Published:** 2021-08-09

**Authors:** Nadeem Ahmad Sheikh, Dennis Ling Chuan Ching, Ilyas Khan, Hamzah Bin Sakidin, Muhammad Jamil, Hafiz Usman Khalid, Nisar Ahmed

**Affiliations:** 1 Fundamental and Applied Sciences Department, Universiti Teknologi PETRONAS Perak, 32610 Seri Iskandar, Malaysia; 2 Department of Mathematics, College of Science Al-Zulfi, Majmaah University, Al-Majmaah, 11952 Saudi Arabia; 3 Department of Geosciences, Universiti Teknologi PETRONAS, 32610 Bandar Seri Iskandar, Perak Malaysia; 4 Department of Earth Sciences, COMSATS University Islamabad, Abbottabad Campus, Abbottabad, 22060 Pakistan; 5 Department of Mechanical Engineering, Universiti Teknologi PETRONAS, 32610 Bandar Seri Iskandar, Perak Malaysia

**Keywords:** Mathematics and computing, Nanoscience and technology, Physics

## Abstract

The present work used fractional model of Casson fluid by utilizing a generalized Fourier’s Law to construct Caputo Fractional model. A porous medium containing nanofluid flowing in a channel is considered with free convection and electrical conduction. A novel transformation is applied for energy equation and then solved by using integral transforms, combinedly, the Fourier and Laplace transformations. The results are shown in form of Mittag-Leffler function. The influence of physical parameters have been presented in graphs and values in tables are discussed in this work. The results reveal that heat transfer increases with increasing values of the volume fraction of nanoparticles, while the velocity of the nanofluid decreases with the increasing values of volume fraction of these particles.

## Introduction

In many engineering and industrial sectors, heat transport is an essential technical subject and becomes a challenge for engineers and manufacturers. To overcome this challenge, the one approach is to surge the available surface area of heat exchange. This technique leads to an unrealistic and unacceptable methodology for the increase in heat transfer in the heat managing systems. Engineers and industrialists face this issue due to the poor thermo-physical properties of conventional fluids such as water, alcohol, ethylene glycol, and oil. Therefore, there is an imperative demand to enhance the thermal conductivity of such fluids to overcome heat transport problems^1^. Nanofluids are used in different engineering and industrial sectors to overcome the heat transfer problems in the conventional fluids. Casson fluid is one on the important industrial fluids as it has tremendous properties and applications.

### Casson nanofluid

In many engineering and industrial sectors, heat transport is an essential technical subject and becomes a challenge for engineers and manufacturers. To overcome this challenge, one of the approaches is to surge the available surface area of heat exchange. This technique leads to an unrealistic and unacceptable methodology for the increase in heat transfer in the heat managing systems. Engineers and industrialists face this issue due to the poor thermo-physical properties of conventional fluids such as water, alcohol, ethylene glycol, and oil. Therefore, there is an imperative demand to enhance the thermal conductivity of such fluids to overcome heat transport problems^1^. Nanofluids are considered excellent solutions to this problem. The suspension of nanometer-sized metal oxides, metals, polymers, carbon nanotubes, or even silica particles is dispersed in conventional fluids^2^. This idea of applying the nanofluids was initially proposed by Choi^3^ in 1995. The high thermal conductivity of nanofluids is an essential mechanism for reducing the clogging process on the walls of heat transfer devices, increased energy efficiency, better performance, and low-cost alternative^4^. Souayeh et al.^5^ examined the Casson nanofluid as a cooling and friction-controlling agent in their investigation. The Prandtl boundary layer equations are solved using the numerical technique with similarity variables. The electrically conducted flow of Casson nanofluid on a solid sphere was analyzed by Alwawi et al.^6^ using the Keller-box method for obtaining the numerical solutions. They have chosen Sodium Alginate as a base fluid and studied the effects of three nanoparticles Titanium dioxide (TiO_2_), Silver (Ag), and Graphite oxide (GO). Ethylene glycol-based nanofluid was reviewed by Saqib et al.^7^ through exact solutions using the integral transformation. They have developed a Casson nanofluid model utilizing the Tiwari and Das model and considered the Molybdenum disulfide as a nanoparticle. Miles and Bessaïh^8^ studied the heat transfer and entropy generation in the nanofluids, considering the flow in a circular annulus embedded in a porous media. The applications of nanofluids in the cooling of electronic systems were discussed by Aglawe, et al.^9^; they have also discussed the processes of preparation and some challenges. Tlili^10^ studied the influence of thermal conductivity on the nanofluids' thermal and physical properties and rheology. Archana, et al.^11^ presented numerical solutions for Casson nanofluid's incompressible and squeezed flow using the Range-Kutta-Fehlberg scheme. The time-dependent flow of Casson nanofluid with radiative heat transfer was studied by Reddy, et al.^12^. They have revealed that the rate of cooling is increased for higher values of the unsteadiness parameter. Lokesh, et al.^13^ discussed the three-dimensional flow of Casson nanofluid and obtained the numerical solutions using the fourth-order Runge–Kutta integration scheme.

### MHD flow of Casson fluid in a porous media

Computational analysis of temperature velocities and skin friction coefficient for an incompressible Casson fluid was examined in a porous media with magnetohydrodynamic (MHD) boundary layer conditions. The temperature increases with an increase in the heat generation parameter, and the higher Casson fluid parameter is associated with the skin friction coefficient^14^. The mathematical investigation of Casson fluid's heat-absorbing and chemically reacting flow (clay or drilling mud) was examined both on a flat plate and vertical cone with a non-Darcy porous medium. The attributes of moving fluid were analyzed, which influence the concentration, velocities, temperature parameters, and average skin-fraction values^15^. The behavior of the MHD flow of Casson fluid over a vertical plate was studied under constant temperature and wall shear conditions in the porous media. The velocity values are higher in the proximity of the plate with higher values of the Casson parameter while the velocity decreases away from the plate. The velocity has inversely related to shear wall stress, while it is directly related to the magnetic parameter^16^. The non-Newtonian flow of Casson fluid on an oscillating plate under the constant wall temperature by applying the Laplace transformation. The velocity field is reduced under the effect of the slip parameter^17^. The numerical analysis of heating and viscous effects under the constant temperature condition was discussed with homotopy solutions. The skin friction coefficient is inversely related to the Hartman number and the Casson parameter^18^. The homotopy analysis method (HAM) also verifies that the parameters of thermal conduction and viscosity for an incompressible Casson fluid are a linear function of temperature in the boundary layer conditions. An increase in the viscosity of Casson fluid results in a reduction of temperature with higher values of the velocity profile. The rate of heat transfer is significantly decreased in the presence of the magnetic field^19^. The phenomenon of heat generation in Casson fluid flow with temperature and concentration parameters was discussed by applying the fractional derivatives. The variation in velocity is associated with time values, as evident from exact solutions. Temperature and velocities are positively linked with the heat generation parameter, while fluid velocity is inversely related to the chemical reaction parameter^20^. The Lagrangian equation computationally analyzed the dynamics of submarine debris flow in viscoplastic fluids to compare various rheological models. The downslope movement of high-density fluid was discussed, keeping the fluid volume constant to describe the transition of fluid between viscous and plastic nature of flow^21^. The numerical analysis of viscosity and yield stress parameters for the rheology of submarine debris flows was incorporated using the plastic Bingham model. The yield surface is widely determined by the shear rate and viscosity of fluid^22^. The flow of MHD Casson fluid in a non-Darcy porous media was discussed with the transformation of the boundary layer equation to the differential equation. Magnetic and Casson parameters significantly influence the concentration, velocity, concentration, and skin friction. The higher values of temperature and concentration are directly related to the magnetic parameter. Casson parameter is directly associated with skin friction, while the magnetic parameter is inversely correlated with skin friction^23,24^. The flow of viscoelastic incompressible fluid through a uniform magnetic field over an infinite accelerated plate through a porous medium. Laplace transformation technique (LTT) was applied to study the velocity parameter and skin friction. The velocity of fluid has positively influenced by elasticity and permeability, while skin friction also increases with an increase in medium permeability^24^. A Caputo fractional model of MHD Casson fluid flowing in a channel was numerically analyzed by applying Fourier and Laplace transformations. The Casson fluid behaves like a Newtonian fluid by increasing the values of the Casson parameter^25^. The numerical study of heat transfer and mass concentration of the MHD flow of nanofluid embedded in a porous media was discussed by Qureshi, et al.^26^, considering the Soret and Dufour effects. Saqib, et al.^27^ obtained exact solutions for the MHD flow of nanofluid. They have discussed the impact of different shapes of nanoparticles in their study. Some other interesting and essential for MHD flow of Casson fluid are discussed in^28–33^ and the reference therein.

### Effect of nanofluids on the properties of engine oil

**Table:** 

S. no.	Research article/authors	Material	Properties
1	Eswaraiah et al.^34^	Ultra-thin Graphene	(a) Graphene was prepared by solar exfoliation of graphite oxide, which removed almost 97% oxygen and made it hydrophobic (b) It can be added in a certain amount to improve base oil properties, including the coefficient of friction, load-bearing capacity, and the wear scar diameter (WSR) (c) When the concentration is around 0.025 mg/mL, load-bearing capacity increases while WSR and friction decrease (d) The absence of carboxyl and epoxide functional groups proved the hydrophobicity of graphene in FTIR spectra
2	Wu and Kao^35^	TiO_2_ nanofluid	(a) Gelation formed by using TiO_2_ in ethylene glycol (b) Friction force was reduced by using TiO_2_ nanoparticle in paraffin oil with conventional engine oil (c) Particle size has a direct relation with the coefficient of friction. 120 nm gave better results as compared to 220 nm
3	Liu et al.^36^	Carbon nanotubes (CNTs)	(a) The upgrading of thermal conductivity by adding CNTs into ethylene glycol and synthetic engine were studied (b) Thermal conductivity of CNT-ethylene glycol suspension enhanced by 12.4% up to volume fraction of 0.01 (c) Thermal conductivity of CNT-synthetic oil suspension enhanced by 30% up to volume fraction of 0.02 (d) The addition of CNTs into the base fluid formed a three-dimensional network that facilitates thermal transport (e) Fibers like CNTs were seen by the SEM and TEM images
4	Sidik et al.^37^		(a) Enhanced thermal conductivity and heat transfer was achieved by dispersing nanofluids in engine oil (b) The performance of the cooling system can be attained at a low volume fraction of nanoparticles (< 1%)
5	Zhang et al.^38^	Nano-graphite	(a) Nano-graphite was added to the heavy-duty diesel engine, and its performance was investigated (b) Around a 3% volume fraction of nano-graphite increased the cooling effect up to 15%
6	Mohammadi et al.^39^	γ-Al_2_O_3_, CuO	(a) γ-Al_2_O_3_ and CuO nanoparticles were used to enhance the thermal conductivity and heat capacity (b) Thermal conductivity was increased while heat capacity decreased when the concentration of nanoparticles increased (c) CuO increased the thermal conductivity by up to 8% while γ-Al_2_O_3_ just 5%
7	Vasheghani^40^	α-Al_2_O_3_, γ-Al_2_O_3_ & AlN	(a) Three types of Al nanoparticles were added in engine oil to enhance the thermal conductivity (b) Improvement of thermal conductivity by AlN was exceptionally better as compared to other components (c) The addition of 3% nanoparticles enhanced the property by 75.2% for AlN, followed by γ-Al_2_O_3_(20 nm) and α-Al_2_O_3_(20 nm) with 37.49% and 31.47% respectively
8	Ettefaghi et al.^41^	CuO	(a) Different concentrations of CuO nanoparticles (0.1, 0.3 & 0.5%) were added in engine oil to study its effect on thermal conductivity, flash, and pour point (b) Thermal conductivity and flash point increased by 3% and 7.5% by adding 0.1% volume fraction of CuO (c) The pour point has more value at 0.2% as compared to other concentrations
9	Wu et al.^42^	CuO, TiO_2_, nanodiamond	(a) Nanoparticles were added to the engine oil and base oil to study the tribological properties (b) The addition of CuO reduced the friction coefficients by 18.4% and 5.8% in engine and base oil, respectively (c) The sphere-like nanoparticles reduced the friction while the anti-wear mechanism was due to the deposition of nanoparticles on CuO worn surface
10	Ali et al.^43^	MoS_2_	(a) Heat transfer and lubrication properties were enhanced by adding MoS_2_ nanoparticles of different shapes (platelet, blade, cylindrical & bricks) (b) The heat transfer rate of blade-shaped nanoparticles enhanced by 7.87%, 9.64%, 14.33%, and 18.95% as compared to platelet, cylinder, and brick-shaped nanoparticles (c) Platelet shaped nanoparticles improved the convection heat transfer by 3.42%, 6.80%, 10.16% and 13.51%
11	Qiu^44^	Ni nanoparticles	(a) The load-carrying capacity was improved by the addition of Ni nanoparticles (b) Lower concentrations of Ni particles gave a better anti-wear performance, below 1% (c) The value of the friction coefficient is smaller when the concentration is between 0.2 and 0.5
12	Wong and Leon^45^	Al nanoparticles	(a) The addition of nanoparticles with diesel fuel increased the total combustion heat (b) The concentration of smoke and nitrous oxide decreased in the emission
13	Asadi and Pourfattah^46^	ZnO, MgO	(a) The viscosity and thermal conductivity have been studied over the temperature range (15–55 °C) and concentration (0.125–1.5%) (b) Thermal conductivity and viscosity showed an increasing trend as the temperature and concentration increased (c) The maximum enhancement was 28% and 32% for ZnO and MgO, respectively (d) The increase in dynamic viscosity took place at 55 °C and 1.5% by just over 124% and 75% for ZnO and MgO, respectively (e) None of these fluids are suitable for the laminar flow regime
14	Hu et al.^47^	Graphite nanoparticles	(a) Three critical properties were studied, including temperature, particle volume fraction, and the shear rate (b) Temperature behaved as an essential factor affecting viscosity as compared to volume fraction (c) The nanofluid behaved as a Newtonian (constant viscosity) if the shear rate is 17–68 s^−1^, but it gave non-linear behavior in the case of 667–3333 s^−1^
15	Soltani et al.^48^	WO_3_, MWCNTs	(a) The effects of volume fraction and temperature were studied on WO3/oil and MWCNT/oil (b) Volume fraction has a more significant effect on thermal conductivity than temperature, but both have a direct relation with conductivity (c) The maximum enhancement of thermal conductivity was at 60 °C and 0.6%
16	Esfe and Esfandeh^49^	ZnO-MWCNT	(a) MWNCT-ZnO (20–80%) has been added in 5W30 engine oil and their affect were studied on different VFs (0.05, 0.1, 0.25, 0.5, 0.75 and 1%) and temperatures (5–55 °C) (b) The mentioned nanofluid behaved as a non-Newtonian fluid, and the viscosity has decreased by increasing shear rate (c) The viscosity had a linear relationship with the VFs but non-linear with temperature
17	Liu et al.^50^	TiO_2_/Ag, Al_2_O_3_/Ag	(a) Both of these hybrid nanofluids behaved as shear-thinning fluid because viscosity decreases by increasing the shear rate (b) The viscosity and volume fraction sa linear relation with the hybrid nanofluids (c) The hybrid nanofluid containing the nanoparticles with different morphologies gave a low viscosity rate
18	Yesawani et al.^51^	Al_2_O_3_	(a) The addition of Al_2_O_3_ nanoparticles in 10W30 engine oil were studied based on viscosity and thermal conductivity (b) At higher concentrations, the viscosity has decreased (c) The reduction of thermal conductivity was different at different values of volume fractions and temperatures (d) The viscosity and thermal conductivity decreased by a maximum of 82.9% and 2.12% at 30 °C, 80.3%, and 3.5% at 60 °C, 80.5% and 5.12% at 80 °C respectively
19	Esfe et al.^52^	MWCNT-ZnO	(a) Addition of MWCNT-ZnO (1:4) to 5W50 engine oil and its lubrication properties were studied at different VFs (0.05, 0.1, 0.25, 0.5, 0.75, and 1%) and temperatures (5–55 °C) (b) The heat transfer rate was enhanced within 35–55 °C and at a VF less than 0.25%; it has a considerable effect on the performance of the car engine (c) The decrement of viscosity up to 9% achieved at a VF of 0.05% at 5 °C and shear rate of 666.5 s^−1^ (d) In hybrid nanofluid, the less dependency of viscosity on temperature proved better lubrication properties at higher temperatures
20	Yang et al.^53^	ZnO	(a) The stability of these nanoparticles was studied at various volume fractions and temperatures (b) The thermal conductivity increased with temperature and VFs (c) The maximum enhancement was obtained by 8.74% at VF and temperature of 1.5% at 55 °C respectively (d) The thermal performance of lubricant is better at high temperatures

### Fractional calculus

In the logic we differentiate or integrate a function once, twice, or whole number of times, differentiation and integration are normally considered as discrete processes in general. In some instances, though, the assessment of a non-integer order derivative is helpful. The definition of fractional computation is not new. In a letter to L'Hospital in 1695 Leibniz created an opportunity to generalize differentiation to non-integer order^54^. These, however, were Liouville, Abel, Heaviside, and Riemann's contributions which progressed fractional derivatives theory^55–58^. The fractional calculus provides more general and precise models of physical systems than ordinary calculus in many fields, for example chemistry, mechanics and biotechnology^59–62^. Fractional derivatives are also used for mathematical modeling of electric circuits, electromagnet theory and fractal theory^63–65^.

## Mathematical modelling

**Figure 1:**
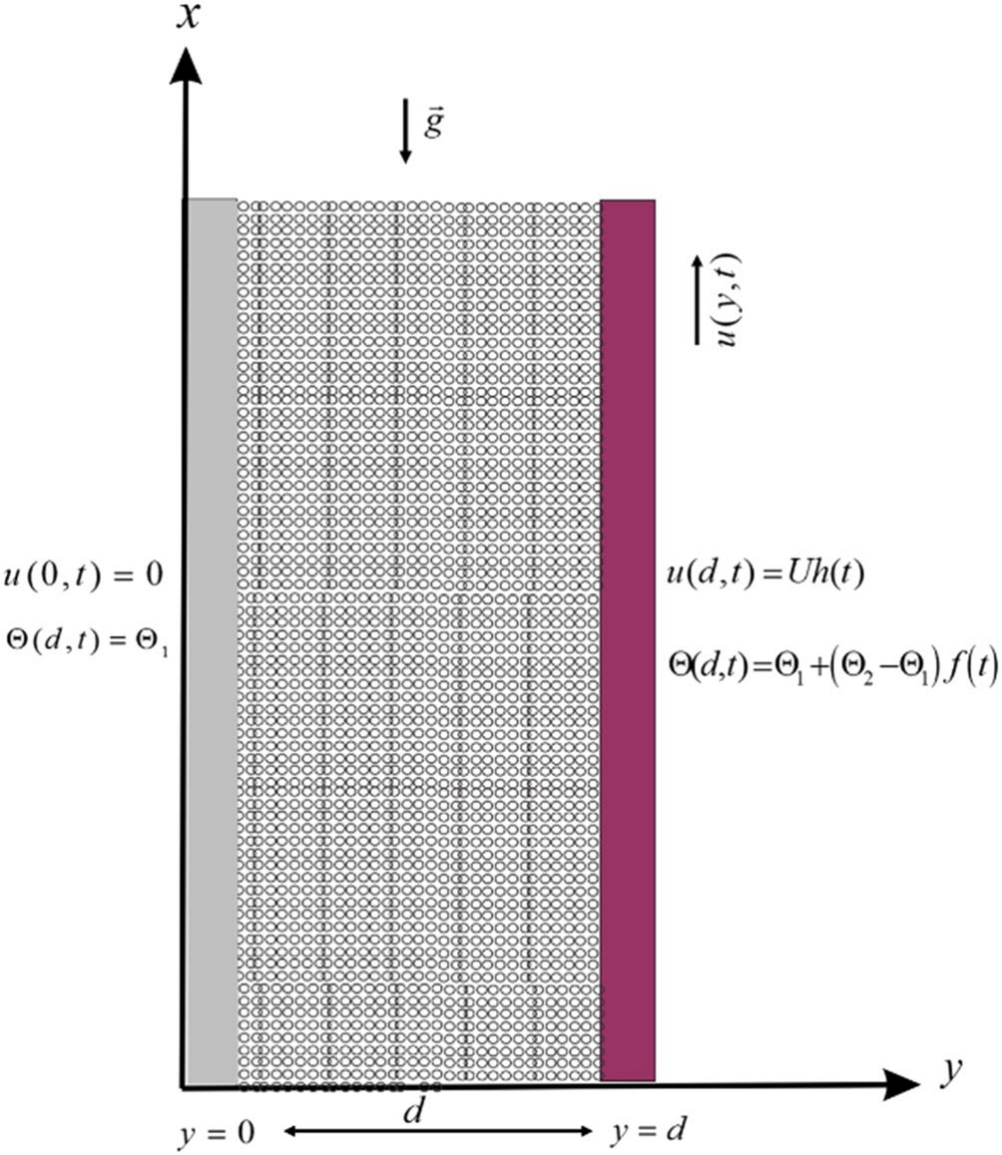
Schematic diagram.

We have considered the motion of Casson nanofluid is a vertical channel embedded in a porous media. The flow is assumed to be in the direction of $x$-axis while the $y$-axis is taken perpendicular to the plates. With ambient temperature $\Theta_{1}$, both the fluid and plates are at rest when $t \le 0$. At $t = 0^{ + }$, the plate at $y = d$ begin to move in its plane with velocity $Uh(t)$ as shown in Fig. 1. At $y = d$, the plate temperature level raised to $\Theta_{1} + \left( {\Theta_{2} - \Theta_{1} } \right)f\left( t \right)$ with time $t.$

We suppose that the rheological equation for an incompressible Casson fluid is^66,67^:

1 $$ \begin{array}{*{20}c} {\tau_{ij} = \left\{ {\begin{array}{*{20}l} {2\left( {\mu_{\gamma } + \tfrac{{p_{y} }}{{\sqrt {2\pi } }}} \right)\,e_{ij} ,} \hfill & {\pi > \pi_{c} ,} \hfill \\ {2\left( {\mu_{\gamma } + \tfrac{{p_{y} }}{{\sqrt {2\pi_{c} } }}} \right)\,e_{ij} ,} \hfill & {\pi_{c} < \pi .} \hfill \\ \end{array} } \right.} \\ \end{array} $$

The free convection flow of Casson nanofluid along with heat and mass transfer and using the well-known Boussinesq's approximation is governed by the following partial differential equations^68,69^:

2 $$ \rho_{nf} \frac{\partial u(y,t)}{{\partial t}} = \mu_{nf} \left( {1 + \frac{1}{{\gamma_{C} }}} \right)\,\frac{{\partial^{2} u(y,t)}}{{\partial y^{2} }} - \sigma_{nf} B_{0}^{2} u(y,t) - \left( {1 + \frac{1}{{\gamma_{C} }}} \right)\frac{{\mu_{nf} }}{k}u(y,t) + \left( {\rho \beta_{\Theta } } \right)_{nf} g(\Theta - \Theta_{1} ), $$

3 $$ \left( {\rho c_{p} } \right)_{nf} \frac{\partial \Theta (y,t)}{{\partial t}} = - \frac{\partial q(y,t)}{{\partial y}}, $$

4 $$ q(y,t) = - k_{nf} \frac{\partial \Theta (y,t)}{{\partial y}}, $$

For the properties of the nanofluids with a subscript $\left( {nf} \right)$, refer to^70^. The thermophysical properties of nanoparticles and base fluid are given in Table 1.

**Table 1:** Thermophysical properties of nanoparticles and base fluid.

Properties	Engine oil	Cadmium telluride (CdTe)
$\rho \left( {{\text{kg}}\;{\text{m}}^{ - 3} } \right)$	863	5855
$Cp\left( {{\text{J}}\;{\text{kg}}^{ - 1} \;{\text{K}}^{ - 1} } \right)$	2048	209
$k\left( {{\text{Wm}}^{ - 1} \;{\text{K}}^{ - 1} } \right)$	0.1404	7.5
$\beta \times 10^{ - 5} \left( {{\text{K}}^{ - 1} } \right)$	0.00007	0.00005
$\sigma \left( {{\text{Sm}}^{ - 1} } \right)$	0.0000055	0.0000007

In the dimensionless form the initial and boundary conditions are:

5 $$ \left. {\begin{array}{*{20}l} {u(y,\,0) = 0,} \hfill & {\Theta (y,\,0) = \Theta_{1} ,} \hfill \\ {u(0,\,t) = 0,} \hfill & {\Theta (0,\,t) = \Theta_{1} ,} \hfill \\ {u(d,\,t) = Uh(t),} \hfill & {\Theta (d,\,t) = \Theta_{1} + \left( {\Theta_{2} - \Theta_{1} } \right)f\left( t \right),} \hfill \\ \end{array} } \right\}. $$

Introducing the following dimensionless variables:

$$ v = \frac{u}{U}, \,\xi = \frac{y}{d}, \,\tau = \frac{{\nu_{f} }}{{d^{2} }}t, \,\theta = \frac{{\Theta - \Theta_{1} }}{{\Theta_{2} - \Theta_{1} }}, \,\delta = \frac{qd}{{k_{f} \left( {\Theta_{2} - \Theta_{1} } \right)}},\, \, \,f(\tau ) = f\left( {\frac{{d^{2} }}{{\nu_{f} }}t} \right),\,\,h(\tau ) = h\left( {\frac{{d^{2} }}{{\nu_{f} }}t} \right), $$

into Eqs. (2), (3), (4) and (5) we get:

6 $$ \frac{\partial v(\xi ,\tau )}{{\partial \tau }} = \beta_{1} \frac{{\partial^{2} v(\xi ,\tau )}}{{\partial \xi^{2} }} - \beta_{2} v(\xi ,\tau ) + \beta_{3} \theta (\xi ,\tau ), $$

7 $$ \frac{\partial \theta (\xi ,\tau )}{{\partial \tau }} = - \frac{1}{{\vartheta_{5} \Pr }}\frac{\partial \delta (\xi ,\tau )}{{\partial \xi }}, $$

8 $$ \delta (\xi ,\tau ) = - \vartheta_{6} \frac{\partial \theta (\xi ,\tau )}{{\partial \xi }} $$

9 $$ \left. {\begin{array}{*{20}l} {v(\xi ,\,0) = 0,} \hfill & {\theta (\xi ,\,0) = 0,} \hfill \\ {v(0,\,\tau ) = 0,} \hfill & {\theta (0,\,\tau ) = 0,} \hfill \\ {v(1,\,\tau ) = h(\tau ),} \hfill & {\theta (1,\,\tau ) = f(\tau ),} \hfill \\ \end{array} } \right\}, $$

where $M = \tfrac{{\sigma_{f} B_{0}^{2} d^{2} }}{{\mu_{f} }}$ is the Hartman number, $K = \frac{kU}{{\nu_{f} d}}$ is the porous media parameter, $\;Gr = \tfrac{{gd^{2} \beta_{\Theta } }}{{\nu_{f} U}}\left( {\Theta_{2} - \Theta_{1} } \right)$ is the thermal Grashof number, and $\Pr = \tfrac{{\left( {\rho c_{p} } \right)_{f} \upsilon_{f} }}{{k_{f} }}$ is the Prandtl number.

### Fractional model

To develop a fractional model for the mentioned flow problem, the generalized Fourier’s law is used as under:

10 $$ \delta (\xi ,\tau ) = - {}^{C}\wp_{\tau }^{1 - \alpha } \left( {\frac{\partial \theta (\xi ,\tau )}{{\partial \xi }}} \right);\quad 0 < \alpha \le 1, $$

where $^{C} \wp_{\tau }^{\alpha } \left( . \right)$ is the Caputo time Fractional Operator and is defined by

11 $$ {}^{C}\wp_{t}^{\alpha } r(y,t) = \frac{1}{{\Gamma \left( {1 - \alpha } \right)}}\int\limits_{0}^{t} {\mathop r\limits^{ \cdot } } (y,s)(t - s)^{ - \alpha } ds = \eta_{\alpha } (t)*\mathop r\limits^{ \cdot } (y,t);\quad 0 < \alpha \le 1, $$

here $\eta_{\alpha } (t) = \frac{{t^{ - \alpha } }}{{\Gamma \left( {1 - \alpha } \right)}}$ is the singular Power-law kernel.

Furthermore,

12 $$ L\left\{ {\eta_{\alpha } (t)} \right\} = \frac{1}{{s^{1 - \alpha } }},\,\left\{ {\eta_{1 - \alpha } *\eta_{\alpha } } \right\}\left( t \right) = 1,\,\eta_{0} (t) = L^{ - 1} \left\{ \frac{1}{s} \right\} = 1,\,\eta_{1} (t) = L^{ - 1} \left\{ 1 \right\} = \zeta (t), $$

here $L\left\{ . \right\}$ is the Laplace transform, $\zeta (.)$ is the Dirac’s delta function and $s$ is the Laplace transform parameter.

Using the above properties and the second form Eq. (11), it is convenient to show that

13 $$^{C} \wp_{t}^{0} r(y,t) = r(y,t) - r(y,0),\, $$

14 $$^{C} \wp_{t}^{1} r(y,t) = \frac{\partial r(y,t)}{{\partial t}} $$

Utilizing the definition of Caputo time fractional operator for Eq. (7) Using Eqs. (8), (10) and (11) we arrived at:

15 $$ \frac{\partial \theta (\xi ,\tau )}{{\partial t}} = \beta_{4} {}^{C}\wp_{\tau }^{1 - \alpha } \left( {\frac{{\partial^{2} \theta (\xi ,\tau )}}{{\partial^{2} \xi }}} \right), $$

To obtain the more suitable form of the Eq. (15) we recall the time fractional integral operator

16 $$ \Im_{t}^{\alpha } r(y,t) = \left( {\eta_{1 - \alpha } *r} \right)(t) = \frac{1}{\Gamma \left( \alpha \right)}\int\limits_{0}^{t} {r(y,s)(t - s)^{\alpha - 1} } ds. $$

This is the inverse operator of the derivative operator $^{C} \wp_{t}^{\alpha } \left( . \right)$. Using the properties from Eq. (12) we have

17 $$ \begin{aligned} & \left( {\Im_{t}^{\alpha } \circ {}^{C}\wp_{\tau }^{\alpha } } \right)r\left( {y,t} \right) = \Im_{t}^{\alpha } \left( {{}^{C}\wp_{\tau }^{\alpha } r\left( {y,t} \right)} \right) = \left[ {\eta_{1 - \alpha } *\left( {\eta_{\alpha } *\mathop r\limits^{ \cdot } } \right)} \right]\left( t \right) \\ & \quad = \left[ {\left( {\eta_{1 - \alpha } *\eta_{\alpha } } \right)*\mathop r\limits^{ \cdot } } \right]\left( t \right) = \left[ {1*\mathop r\limits^{ \cdot } } \right](t) = r(y,t) - r(y,0), \\ \end{aligned} $$

18 $$ \Rightarrow \left( {\Im_{t}^{\alpha } \circ {}^{C}\wp_{\tau }^{\alpha } } \right)r\left( {y,t} \right) = r(y,t)\quad if\quad r\left( {y,0} \right) = 0. $$

Using the property, $\Im_{t}^{1 - \alpha } \mathop r\limits^{ \cdot } \left( {y,t} \right) = \left( {\eta_{\alpha } *\mathop r\limits^{ \cdot } } \right)(t) = {}^{C}\wp_{t}^{\alpha } r\left( {y,t} \right),$ Eq. (15) can be written as:

19 $$ {}^{C}\wp_{\tau }^{\alpha } \theta (\xi ,\tau ) = \beta_{4} \frac{{\partial^{2} \theta (\xi ,\tau )}}{{\partial^{2} \xi }}, $$

## Solution of the problem

The derived fractional is solved using the new defined mathematical setting and the integral transforms.

### Energy field

Using the following transformation

20 $$ \chi \left( {\xi ,\tau } \right) = \theta \left( {\xi ,\tau } \right) - \xi f\left( \tau \right), $$

Equation (19) takes the form

21 $$ {}^{C}\wp_{\tau }^{\alpha } \chi (\xi ,\tau ) + \xi {}^{C}\wp_{\tau }^{\alpha } f(\tau ) = \beta_{4} \frac{{\partial^{2} \chi (\xi ,\tau )}}{{\partial^{2} \xi }}, $$

with the corresponding initial and boundary conditions as:

22 $$ \chi (\xi ,0) = 0,\,\,\chi (0,\tau ) = 0,\,\,\chi (1,\tau ) = 0. $$

Applying the Laplace and Fourier sine transform, we get

23 $$ \overline{\chi }_{F} (n,s) = s\overline{f}(s)\frac{{\left( { - 1} \right)^{n} }}{n\pi }\frac{{s^{\alpha - 1} }}{{s^{\alpha } + \beta_{6} }}, $$

inverting the integral transformations of Eq. (23), we have

24 $$ \chi (\xi ,\tau ) = 2\sum\limits_{n = 1}^{\infty } {\frac{{\left( { - 1} \right)^{n} \sin \left( {\xi n\pi } \right)}}{n\pi }} \int\limits_{0}^{\tau } {\mathop f\limits^{ \cdot } } \left( {\tau - t} \right)E_{\alpha ,\alpha - 1} \left( { - \beta_{6} t^{\alpha } } \right)dt, $$

therefore, the solution of the energy equation is

25 $$ \theta \left( {\xi ,\tau } \right) = \chi \left( {\xi ,\tau } \right) + \xi f\left( \tau \right). $$

### Velocity profile

Applying the Laplace and Fourier transforms to Eq. (6) using Eq. (9) we arrived at

26 $$ \begin{aligned} \overline{v}_{F} \left( {n,s} \right) & = \frac{{\left( { - 1} \right)^{n + 1} \overline{h}\left( s \right)}}{n\pi } + \left( {\frac{{\beta_{7} }}{s} + \frac{{\beta_{8} }}{{s + \beta_{5} }}} \right)\frac{{\left( { - 1} \right)^{n} s\overline{h}\left( s \right)}}{n\pi } \\ & \quad + \frac{{\beta_{3} }}{{s + \Re_{1} }}\left( {s\overline{f}(s)\frac{{\left( { - 1} \right)^{n} }}{n\pi }\frac{{s^{\alpha - 1} }}{{s^{\alpha } + \beta_{6} }} + \overline{f}(s)\frac{{\left( { - 1} \right)^{n + 1} }}{n\pi }} \right), \\ \end{aligned} $$

where

$$ \begin{aligned} \beta_{0} & = 1 + \frac{1}{{\gamma_{C} }},\;\;\beta_{1} = \frac{{\vartheta_{2} }}{{\beta_{0} \vartheta_{1} }},\;\;\beta_{2} = \frac{1}{{\vartheta_{1} }}\left( {\vartheta_{3} M + \frac{{\vartheta_{2} }}{{\beta_{0} K}}} \right),\;\;\beta_{3} = \frac{{\vartheta_{4} Gr}}{{\vartheta_{1} }},\;\;\beta_{4} = \frac{{\vartheta_{6} }}{{\vartheta_{5} \Pr }},\;\;\beta_{5} = \beta_{2} + \beta_{1} \left( {n\pi } \right)^{2} , \\ \beta_{6} & = \beta_{4} \left( {n\pi } \right)^{2} ,\;\;\beta_{7} = \frac{{\beta_{2} }}{{\beta_{5} }},\;\;\beta_{8} = \frac{{\beta_{5} - \beta_{2} }}{{\beta_{5} }},\;\;\vartheta_{1} = (1 - \phi ) + \phi \left( {\frac{{\rho_{p} }}{{\rho_{f} }}} \right),\;\;\vartheta_{2} = \frac{1}{{(1 - \phi )^{2.5} }}, \\ \vartheta_{3} & = 1 + 3\frac{(\sigma - 1)\phi }{{(\sigma + 2) - (\sigma - 1)\phi }},\;\;\vartheta_{4} = (1 - \phi ) + \phi \left( {\frac{{\rho_{p} \beta_{\Theta p} }}{{\rho_{f} \beta_{\Phi f} }}} \right),\;\;\vartheta_{5} = (1 - \phi ) + \phi \frac{{\rho_{p} c_{pp} }}{{\rho_{f} c_{pf} }},\;\;\vartheta_{6} = \frac{{k_{nf} }}{{k_{f} }}, \\ \end{aligned} $$

inverting the Laplace and Fourier sine transformations of Eq. (26) we have:

27 $$ \begin{aligned} v\left( {\xi ,\tau } \right) & = h\left( \tau \right)\xi + 2\sum\limits_{n = 1}^{\infty } {\frac{{\left( { - 1} \right)^{n} }}{n\pi }\mathop h\limits^{ \cdot } } \left( \tau \right)*\left( {\beta_{7} H(\tau ) + \beta_{8} \exp \left( { - \beta_{5} \tau } \right)} \right)\sin \left( {\xi n\pi } \right) \\ & \quad + 2\beta_{3} \sum\limits_{n = 1}^{\infty } {\left( {\frac{{\left( { - 1} \right)^{n} }}{n\pi }\exp \left( { - \beta_{5} \tau } \right)*\left( {\int\limits_{0}^{\tau } {\mathop f\limits^{ \cdot } } \left( {\tau - q} \right)E_{\alpha ,\alpha - 1} \left( { - \beta_{6} q^{\alpha } } \right)dq + f\left( \tau \right)} \right)} \right)} \sin \left( {\xi n\pi } \right) \\ \end{aligned} $$

here $H(\tau )$ is the unit step function and $E_{a,b} \left( . \right)$ is the Mittag Leffler function^71^.

### Limiting cases

For $\gamma_{C} \to \infty$ the obtained solution is reduced to the solution calculated by Shao et al.^72^ (for $f_{2} (t) = 0$). This shows the validity of the present solutions. For details, please see Eq. 52 in^72^.

For $\phi = 0$, the solution in Eq. (27) are reduced to the flow of conventional fluid without nanoparticles, Please see^73^ in the absence of mass transfer i.e. $Gm = 0$.

### Nusselt number

Nusselt number is an essential physical quantity, especially for engineers and industrialists. In nondimensional form Nusselt number is given by Eq. (28):

28 $$ Nu = \left. {\frac{{\partial \theta \left( {\xi ,\tau } \right)}}{\partial \xi }} \right|_{\xi = 1} . $$

## Results and discussion

The exact solutions for the MHD flow of Casson nanofluid in a channel embedded in a porous media with heat transfer are obtained in this study. The associated energy equation is fractionalized using generalized Fourier’s law. The obtained exact solutions are plotted through graphs, and the effects of different physical parameters on the flow and heat transfer are presented.

**Figure 2:**
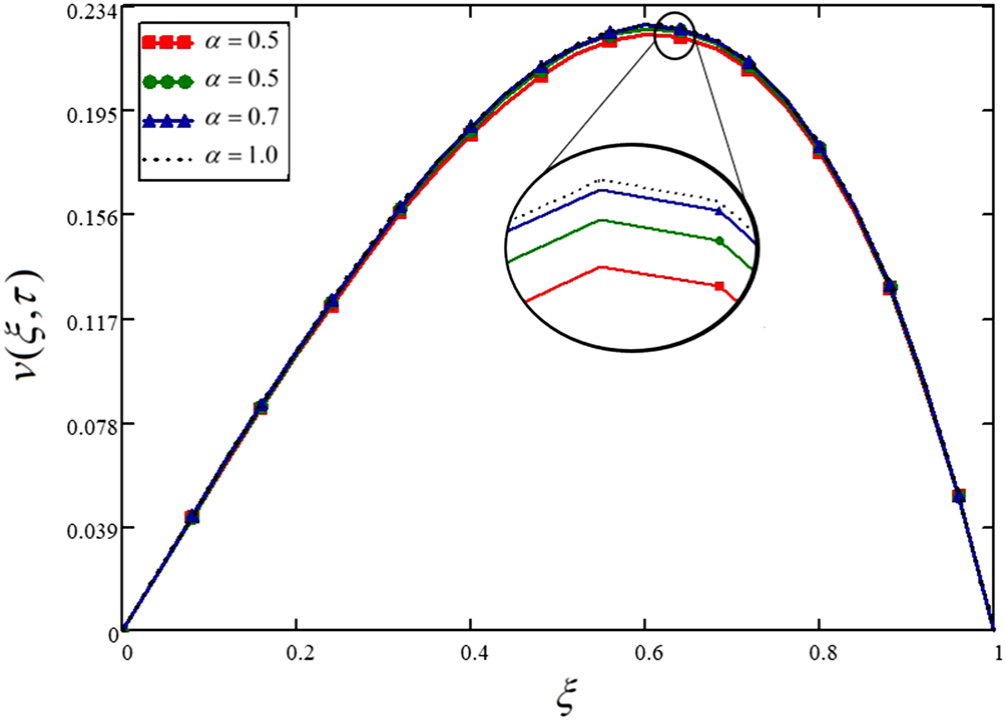
Influence of the fractional parameter on the nanofluid velocity.

The variations in the nanofluid velocity for different values of the fractional parameter are displayed in Fig. 2. From this figure, it is noticed that four different velocity profiles are obtained for four different values of fractional parameter keeping all the other physical parameters constant. This shows that the fractional parameter significantly influences the obtained solutions, even this is not a physical parameter and is a purely mathematical parameter. These variations are due to the memory effect, which cannot be studied through integers order derivatives. These variations are also presented in Table 2 for the ease of numerical and experimental solvers.

**Table 2:** Variations in velocity profile against $\xi$ if $h(\tau ) = 0$ for different values of $\alpha$_._

$\xi$	$v(\xi ,\tau )$ at $\alpha = 0.3$	$v(\xi ,\tau )$ at $\alpha = 0.5$	$v(\xi ,\tau )$ at $\alpha = 0.7$	$v(\xi ,\tau )$ at $\alpha = 1.0$
0	0	0	0	0
0.04	0.021	0.021	0.022	0.022
0.08	0.042	0.043	0.043	0.043
0.12	0.063	0.064	0.064	0.064
0.16	0.083	0.084	0.085	0.085
0.2	0.103	0.104	0.105	0.105
0.24	0.122	0.123	0.124	0.124
0.28	0.14	0.141	0.142	0.142
0.32	0.156	0.158	0.159	0.159
0.36	0.172	0.174	0.175	0.175
0.4	0.186	0.187	0.189	0.189
0.44	0.198	0.2	0.201	0.201
0.48	0.208	0.21	0.211	0.211
0.52	0.216	0.218	0.219	0.219
0.56	0.221	0.223	0.224	0.225
0.6	0.223	0.225	0.226	0.227
0.64	0.222	0.224	0.226	0.226
0.68	0.218	0.22	0.221	0.221
0.72	0.21	0.211	0.212	0.213
0.76	0.197	0.198	0.199	0.2
0.8	0.179	0.181	0.182	0.182
0.84	0.157	0.158	0.158	0.159
0.88	0.128	0.129	0.129	0.13
0.92	0.092	0.093	0.094	0.094
0.96	0.05	0.05	0.051	0.051
1	0	0	0	0

**Figure 3:**
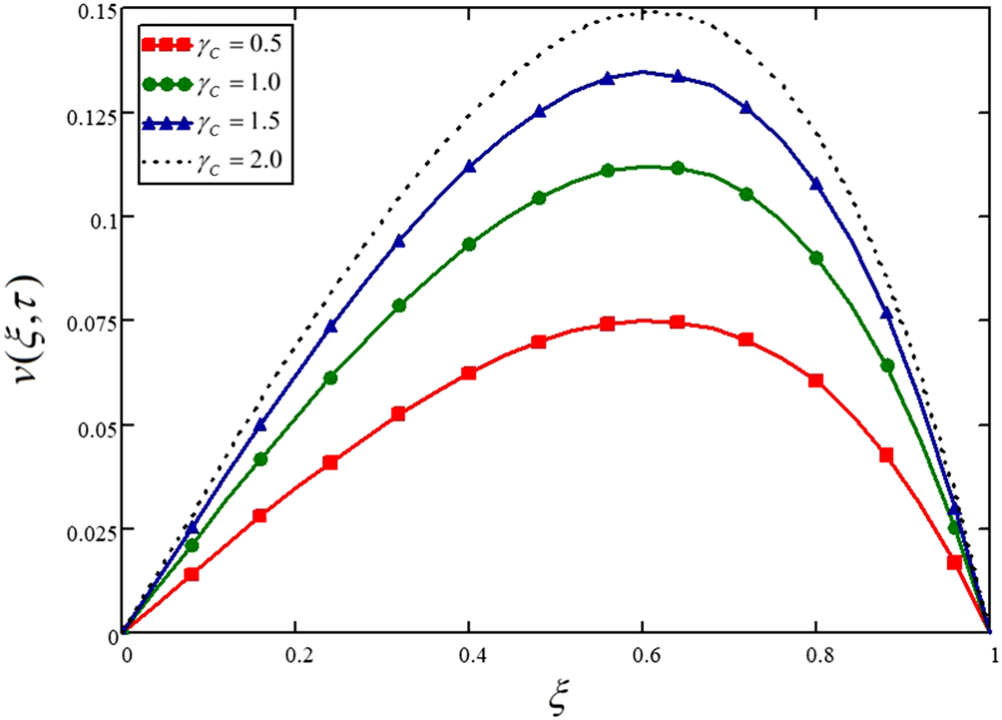
Influence of the Casson fluid parameter on the nanofluid velocity.

An increasing trend is noticed in the velocity of the Casson nanofluid for increasing values of the Casson fluid parameter in Fig. 3 and Table 3. Physically, the viscosity of the fluid is increased for smaller values of the Casson fluid parameter. Another impressive result can be drawn through this graph that Casson fluid is more viscous than Newtonian fluid and when $\gamma_{C} \to \infty$, the fluid behaves like a Newtonian fluid.

**Table 3:** Variations in velocity profile against $\xi$ if $h(\tau ) = 1$ for different values of $\gamma_{C}$_._

$\xi$	$v(\xi ,\tau )$ at $\gamma_{C} = 0.5$	$v(\xi ,\tau )$ at $\gamma_{C} = 2$	$v(\xi ,\tau )$ at $\gamma_{C} = 5$	$v(\xi ,\tau )$ at $\gamma_{C} = 10$
0	0	0	0	0
0.04	7.14e^−3^	0.011	0.013	0.014
0.08	0.014	0.021	0.025	0.028
0.12	0.021	0.032	0.038	0.042
0.16	0.028	0.042	0.05	0.056
0.2	0.035	0.052	0.062	0.069
0.24	0.041	0.061	0.073	0.082
0.28	0.047	0.07	0.084	0.094
0.32	0.053	0.079	0.094	0.105
0.36	0.058	0.086	0.104	0.115
0.4	0.062	0.093	0.112	0.124
0.44	0.067	0.099	0.119	0.132
0.48	0.07	0.104	0.125	0.139
0.52	0.073	0.108	0.13	0.144
0.56	0.074	0.111	0.133	0.148
0.6	0.075	0.112	0.134	0.149
0.64	0.075	0.112	0.134	0.149
0.68	0.073	0.109	0.131	0.146
0.72	0.07	0.105	0.126	0.14
0.76	0.066	0.099	0.119	0.132
0.8	0.06	0.09	0.108	0.12
0.84	0.053	0.079	0.094	0.105
0.88	0.043	0.064	0.077	0.085
0.92	0.031	0.046	0.056	0.062
0.96	0.017	0.025	0.03	0.034
1	0	0	0	0

**Figure 4:**
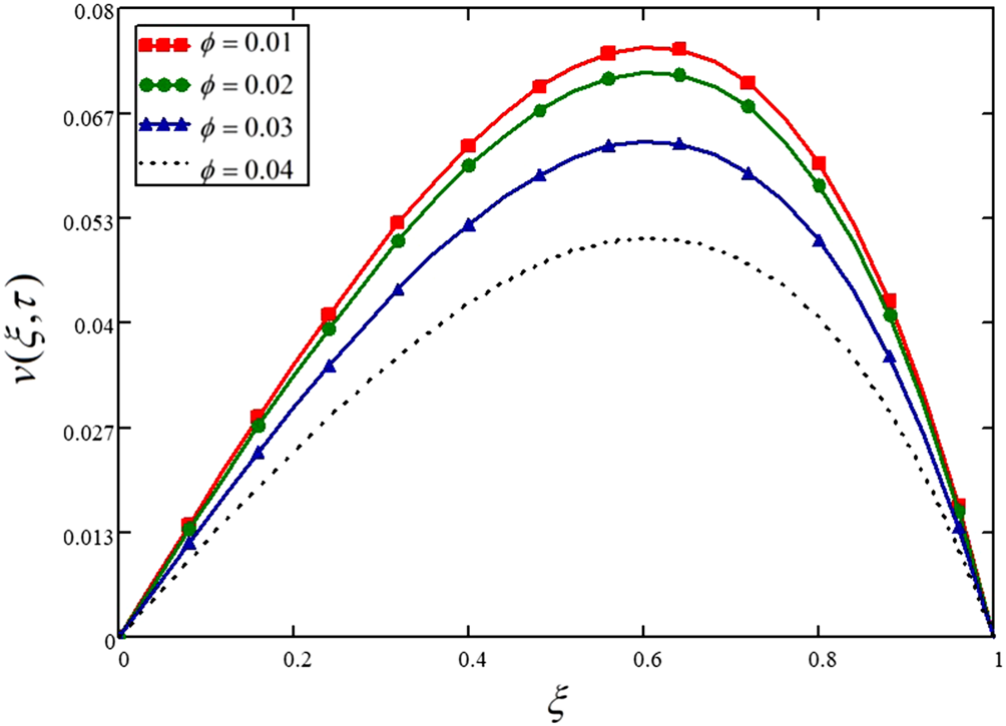
Influence of nanoparticles volume fraction on the nanofluid velocity.

This study considered engine oil as a base fluid and Cadmium Telluride (CdTe) as nanoparticles. Figure 4 is drawn to show the effect of the volume fraction of nanoparticles on the fluid velocity. The fluid velocity is decreasing with the higher values of the volume fraction of nanofluid. This means the fluid will become more viscous with the addition of nanoparticles, and as a result, the lubrication of the engine oil will be improved. For the interest of the readers, Table 4 is also presented for the same phenomenon.

**Table 4:** Variations in velocity profile against $\xi$ if $h(\tau ) = 1$ for different values of $\phi$_._

$\xi$	$v(\xi ,\tau )$ at $\phi = 0.01$	$v(\xi ,\tau )$ at $\phi = 0.02$	$v(\xi ,\tau )$ at $\phi = 0.03$	$v(\xi ,\tau )$ at $\phi = 0.04$
0	0	0	0	0
0.04	7.14e^−3^	6.846e^−3^	6.013e^−3^	4.86e^−3^
0.08	0.014	0.014	0.012	9.684e^−3^
0.12	0.021	0.02	0.018	0.014
0.16	0.028	0.027	0.024	0.019
0.2	0.035	0.033	0.029	0.024
0.24	0.041	0.039	0.035	0.028
0.28	0.047	0.045	0.04	0.032
0.32	0.053	0.05	0.044	0.036
0.36	0.058	0.055	0.049	0.039
0.4	0.062	0.06	0.053	0.042
0.44	0.067	0.064	0.056	0.045
0.48	0.07	0.067	0.059	0.047
0.52	0.073	0.069	0.061	0.049
0.56	0.074	0.071	0.062	0.05
0.6	0.075	0.072	0.063	0.051
0.64	0.075	0.072	0.063	0.051
0.68	0.073	0.07	0.061	0.05
0.72	0.07	0.067	0.059	0.048
0.76	0.066	0.063	0.055	0.045
0.8	0.06	0.058	0.05	0.041
0.84	0.053	0.05	0.044	0.035
0.88	0.043	0.041	0.036	0.029
0.92	0.031	0.03	0.026	0.021
0.96	0.017	0.016	0.014	0.011
1	0	0	0	0

**Figure 5:**
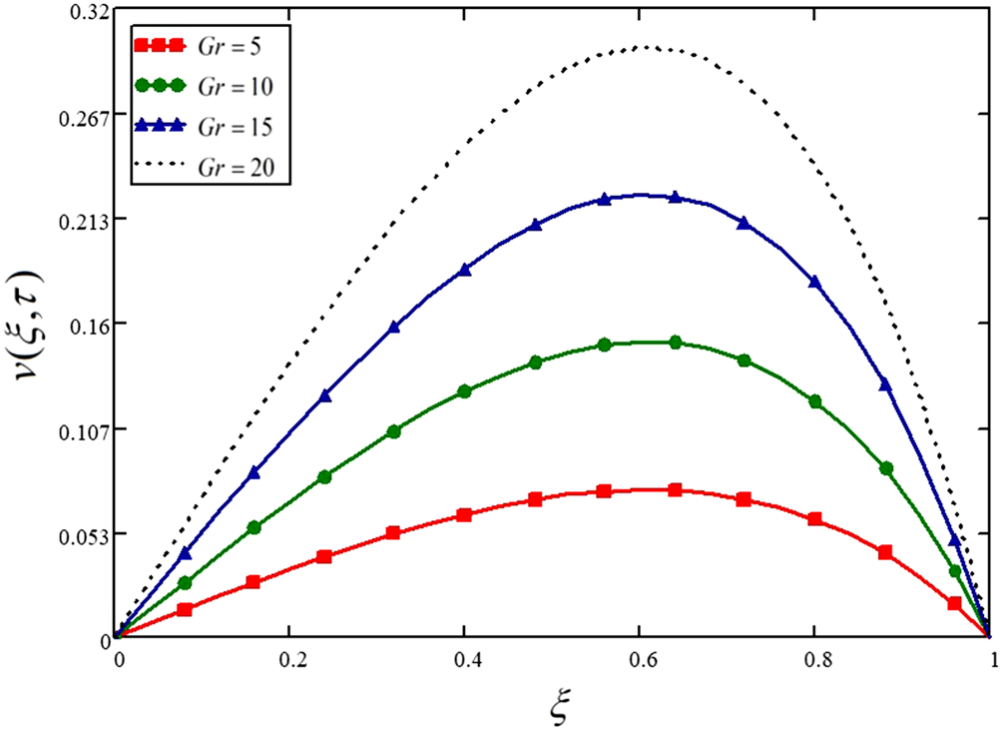
Influence of thermal Grashof number on the nanofluid velocity.

Figure 5 and Table 5 are presented to show the influence of thermal Grashof number on the fluid velocity. Grashof number is the ratio of buoyancy forces to the viscous forces. The greater values of Grashof number means higher buoyancy forces, and hence the velocity is increasing with the higher values of $Gr$.

**Table 5:** Variations in velocity profile against $\xi$ if $h(\tau ) = 0$ for different values of $Gr$_._

$\xi$	$v(\xi ,\tau )$ at $Gr = 5$	$v(\xi ,\tau )$ at $Gr = 10$	$v(\xi ,\tau )$ at $Gr = 15$	$v(\xi ,\tau )$ at $Gr = 20$
0	0	0	0	0
0.04	7.14e^−3^	0.014	0.021	0.029
0.08	0.014	0.028	0.043	0.057
0.12	0.021	0.042	0.064	0.085
0.16	0.028	0.056	0.084	0.112
0.2	0.035	0.069	0.104	0.139
0.24	0.041	0.082	0.123	0.164
0.28	0.047	0.094	0.141	0.188
0.32	0.053	0.105	0.158	0.211
0.36	0.058	0.116	0.174	0.231
0.4	0.062	0.125	0.187	0.25
0.44	0.067	0.133	0.2	0.266
0.48	0.07	0.14	0.21	0.28
0.52	0.073	0.145	0.218	0.29
0.56	0.074	0.149	0.223	0.297
0.6	0.075	0.15	0.225	0.3
0.64	0.075	0.15	0.224	0.299
0.68	0.073	0.147	0.22	0.293
0.72	0.07	0.141	0.211	0.282
0.76	0.066	0.132	0.198	0.265
0.8	0.06	0.12	0.181	0.241
0.84	0.053	0.105	0.158	0.21
0.88	0.043	0.086	0.129	0.171
0.92	0.031	0.062	0.093	0.124
0.96	0.017	0.034	0.05	0.067
1	0	0	0	0

**Figure 6:**
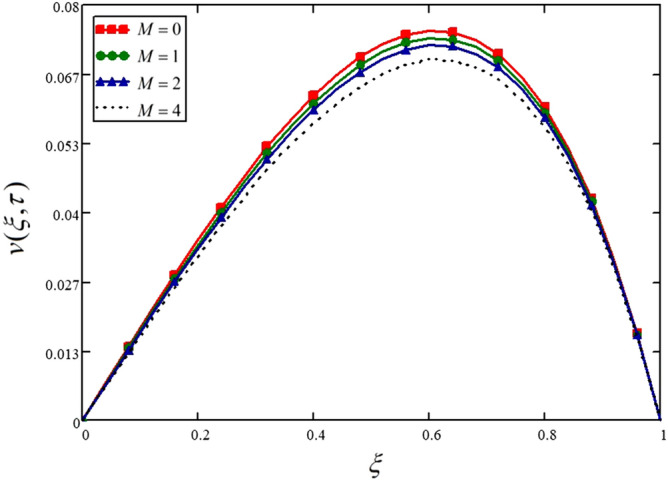
Influence of Hartman number on the nanofluid velocity.

In this study, the MHD flow is considered. The velocity profile shows a decreasing trend for increasing vales of Hartman number in Fig. 6 and Table 6. Physically, higher values of $M$ mean greater Lorentz forces flow opposing forces and control the flow of fluid. In Fig. 6, the velocity profile for non-MHD flow is also drawn when $M = 0$.

**Table 6:** Variations in velocity profile against $\xi$ if $h(\tau ) = 0$ for different values of $M$_._

$\xi$	$v(\xi ,\tau )$ at $M = 0$	$v(\xi ,\tau )$ at $M = 1$	$v(\xi ,\tau )$ at $M = 2$	$v(\xi ,\tau )$ at $M = 4$
0	0	0	0	0
0.04	7.14e^−3^	6.968e^−3^	6.803e^−3^	6.493e^−3^
0.08	0.014	0.014	0.014	0.013
0.12	0.021	0.021	0.02	0.019
0.16	0.028	0.027	0.027	0.026
0.2	0.035	0.034	0.033	0.032
0.24	0.041	0.04	0.039	0.037
0.28	0.047	0.046	0.045	0.043
0.32	0.053	0.051	0.05	0.048
0.36	0.058	0.057	0.055	0.053
0.4	0.062	0.061	0.06	0.057
0.44	0.067	0.065	0.064	0.061
0.48	0.07	0.068	0.067	0.064
0.52	0.073	0.071	0.07	0.067
0.56	0.074	0.073	0.071	0.069
0.6	0.075	0.074	0.072	0.069
0.64	0.075	0.073	0.072	0.069
0.68	0.073	0.072	0.071	0.068
0.72	0.07	0.069	0.068	0.066
0.76	0.066	0.065	0.064	0.062
0.8	0.06	0.059	0.058	0.056
0.84	0.053	0.052	0.051	0.049
0.88	0.043	0.042	0.042	0.04
0.92	0.031	0.031	0.03	0.029
0.96	0.017	0.017	0.016	0.016
1	0	0	0	0

**Figure 7:**
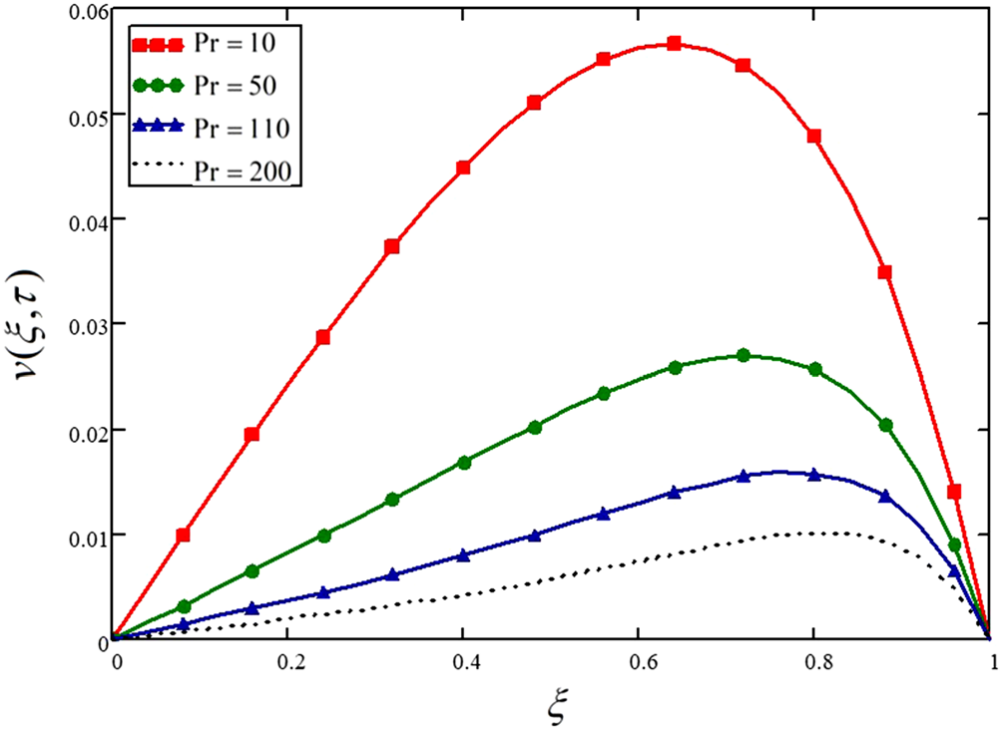
Influence of Prandtl number on the nanofluid velocity.

Prandtl number is the ratio of viscous forces to the thermal forces. The greater values of Prandtl number result in the higher viscous forces and weaker thermal forces and, as a result, decelerate the flow of the nanofluid. This phenomenon is described in Fig. 7 and Table 7.

**Table 7:** Variations in velocity profile against if for different values of.

$$\Pr$$$$\xi$$	$$v(\xi ,\tau )$$ at $$\Pr = 0.7$$	$$v(\xi ,\tau )$$ at $$\Pr = 7$$	$$v(\xi ,\tau )$$ at $$\Pr = 15$$	$$v(\xi ,\tau )$$ at $$\Pr = 21$$
0	0	0	0	0
0.04	4.937e^−3^	1.623e^−3^	7.179e^−4^	3.822e^−4^
0.08	9.85e^−3^	3.251e^−3^	1.441e^−3^	7.676e^−4^
0.12	0.015	4.89e^−3^	2.175e^−3^	1.159e^−3^
0.16	0.019	6.545e^−3^	2.925e^−3^	1.56e^−3^
0.2	0.024	8.22e^−3^	3.695e^−3^	1.974e^−3^
0.24	0.029	9.916e^−3^	4.491e^−3^	2.403e^−3^
0.28	0.033	0.012	5.317e^−3^	2.852e^−3^
0.32	0.037	0.013	6.176e^−3^	3.323e^−3^
0.36	0.041	0.015	7.072e^−3^	3.82e e^−3^
0.4	0.045	0.017	8.004e^−3^	4.346e^−3^
0.44	0.048	0.019	8.972e^−3^	4.902e^−3^
0.48	0.051	0.02	9.971e^−3^	5.492e^−3^
0.52	0.053	0.022	0.011	6.114e^−3^
0.56	0.055	0.023	0.012	6.767e^−3^
0.6	0.056	0.025	0.013	7.444e^−3^
0.64	0.057	0.026	0.014	8.13e^−3^
0.68	0.056	0.027	0.015	8.802e^−3^
0.72	0.055	0.027	0.015	9.42e^−3^
0.76	0.052	0.027	0.016	9.921e^−3^
0.8	0.048	0.026	0.016	0.01
0.84	0.042	0.024	0.015	0.01
0.88	0.035	0.02	0.014	9.458e^−3^
0.92	0.026	0.016	0.011	7.885e^−3^
0.96	0.014	9.013e^−3^	6.539e^−3^	4.946e^−3^
1	0	0	0	0

**Figure 8:**
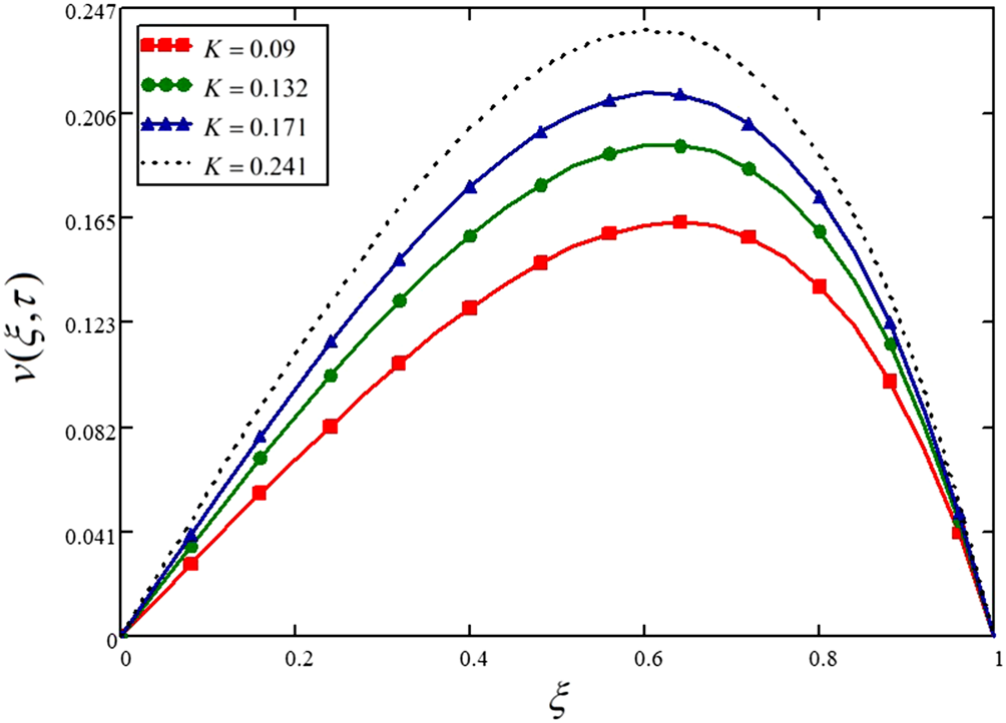
Influence of Darcey number on the nanofluid velocity.

The effect of the Darcey number (Permeability parameter) is shown on the velocity profile in Fig. 8 and Table 8. The velocity is increasing with the increasing values of $K$. The greater values of $K$ means, the higher permeability of the media, and hence the media will allow the fluid to move fast and smoothly.

**Table 8:** Variations in velocity profile against $\xi$ if $h(\tau ) = 0$ for different values of $K$_._

$\xi$	$v(\xi ,\tau )$ at $K = 0.09$	$v(\xi ,\tau )$ at $K = 0.132$	$v(\xi ,\tau )$ at $K = 0.171$	$v(\xi ,\tau )$ at $K = 0.241$
0	0	0	0	0
0.04	0.014	0.018	0.02	0.023
0.08	0.028	0.035	0.04	0.046
0.12	0.043	0.053	0.06	0.068
0.16	0.056	0.07	0.079	0.09
0.2	0.07	0.087	0.098	0.111
0.24	0.083	0.103	0.115	0.132
0.28	0.096	0.118	0.133	0.151
0.32	0.108	0.132	0.149	0.169
0.36	0.119	0.146	0.163	0.185
0.4	0.129	0.158	0.177	0.2
0.44	0.138	0.169	0.188	0.212
0.48	0.147	0.178	0.198	0.223
0.52	0.153	0.185	0.206	0.231
0.56	0.158	0.19	0.211	0.236
0.6	0.162	0.193	0.213	0.238
0.64	0.163	0.193	0.213	0.237
0.68	0.161	0.19	0.209	0.232
0.72	0.157	0.184	0.201	0.222
0.76	0.149	0.174	0.189	0.208
0.8	0.138	0.159	0.173	0.189
0.84	0.122	0.14	0.151	0.165
0.88	0.101	0.115	0.124	0.134
0.92	0.074	0.084	0.09	0.097
0.96	0.041	0.046	0.049	0.052
1	0	0	0	0

**Figure 9:**
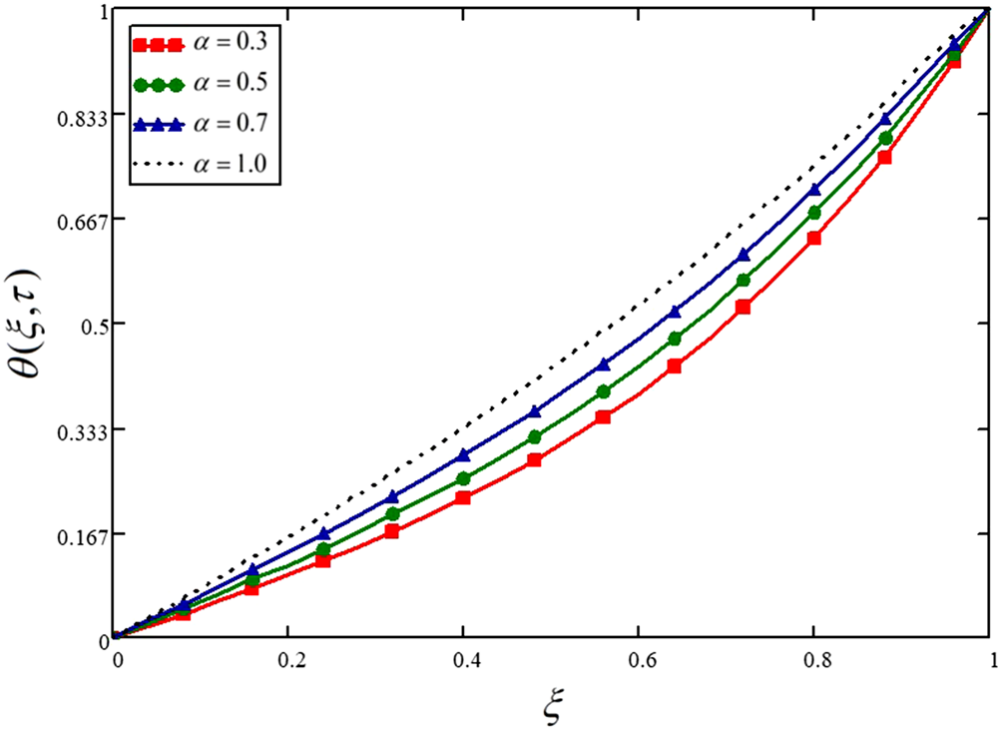
Influence of fractional parameter on the temperature profile.

The variations in the temperature of the nanofluid for different values of the fractional parameter is displayed in Fig. 9 and Table 9. From this figure, it is noticed that four different temperature profiles are obtained for four different values of fractional parameter keeping all the other physical parameters constant. This is showing that the fractional parameter has a significant influence on the obtained solutions; even this is not a physical parameter and is a purely mathematical parameter. These variations are due to the memory effect, which cannot be described through the integer order derivatives model.

**Table 9:** Variations in temperature profile against $\xi$ if $f(\tau ) = 1$ for different values of $\alpha$_._

$\xi$	$\theta (\xi ,\tau )$ at $\alpha = 0.3$	$\theta (\xi ,\tau )$ at $\alpha = 0.5$	$\theta (\xi ,\tau )$ at $\alpha = 0.7$	$\theta (\xi ,\tau )$ at $\alpha = 1.0$
0	0	0	0	0
0.04	0.019	0.023	0.027	0.032
0.08	0.039	0.046	0.054	0.063
0.12	0.059	0.069	0.081	0.095
0.16	0.079	0.093	0.108	0.127
0.2	0.1	0.117	0.137	0.16
0.24	0.122	0.142	0.165	0.193
0.28	0.145	0.168	0.195	0.227
0.32	0.169	0.195	0.226	0.262
0.36	0.194	0.224	0.257	0.297
0.4	0.221	0.254	0.29	0.333
0.44	0.25	0.285	0.324	0.37
0.48	0.281	0.318	0.36	0.408
0.52	0.315	0.354	0.397	0.448
0.56	0.35	0.391	0.436	0.488
0.6	0.389	0.431	0.476	0.529
0.64	0.431	0.474	0.519	0.571
0.68	0.476	0.519	0.563	0.614
0.72	0.525	0.567	0.61	0.658
0.76	0.578	0.619	0.66	0.703
0.8	0.635	0.674	0.712	0.75
0.84	0.697	0.733	0.767	0.8
0.88	0.764	0.795	0.824	0.851
0.92	0.837	0.86	0.884	0.905
0.96	0.915	0.929	0.943	0.958
1	1	1	1	1

**Figure 10:**
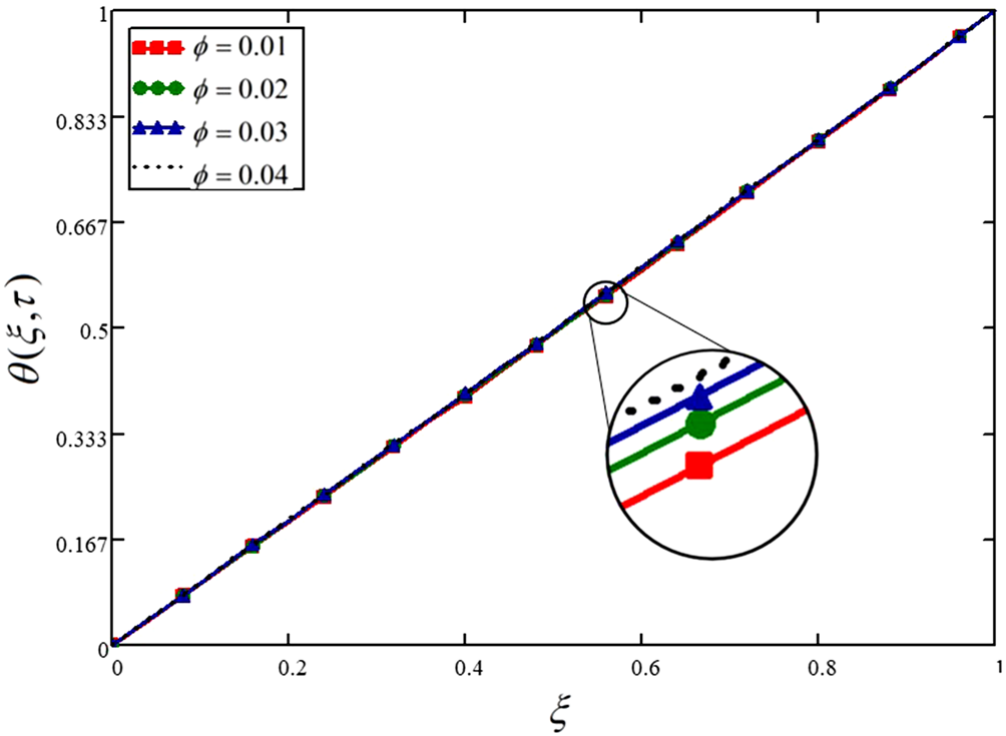
Influence of nanoparticles volume fraction on the temperature profile.

Engine oil as a base fluid and Cadmium Telluride nanoparticles are considered in this analysis. Figure 10 is drawn to show the effect of the volume fraction of nanoparticles on the temperature profile. The temperature is increasing with the increasing values of volume fraction of the nanoparticles. These results are also presented in Table 10.

**Table 10:** Variations in temperature profile against $\xi$ if $f(\tau ) = 1$ for different values of $\phi$_._

$\xi$	$\theta (\xi ,\tau )$ at $\phi = 0.01$	$\theta (\xi ,\tau )$ at $\phi = 0.03$	$\theta (\xi ,\tau )$ at $\phi = 0.03$	$\theta (\xi ,\tau )$ at $\phi = 0.04$
0	0	0	0	0
0.04	0.039	0.039	0.04	0.04
0.08	0.078	0.079	0.079	0.079
0.12	0.117	0.118	0.119	0.119
0.16	0.156	0.157	0.158	0.159
0.2	0.195	0.197	0.198	0.199
0.24	0.234	0.236	0.237	0.238
0.28	0.274	0.276	0.277	0.278
0.32	0.313	0.315	0.317	0.318
0.36	0.352	0.355	0.356	0.358
0.4	0.392	0.394	0.396	0.397
0.44	0.431	0.434	0.436	0.437
0.48	0.471	0.474	0.476	0.477
0.52	0.511	0.514	0.516	0.517
0.56	0.551	0.554	0.556	0.557
0.6	0.591	0.594	0.596	0.597
0.64	0.631	0.634	0.636	0.637
0.68	0.672	0.674	0.676	0.677
0.72	0.712	0.715	0.716	0.718
0.76	0.753	0.755	0.757	0.758
0.8	0.794	0.796	0.797	0.798
0.84	0.835	0.836	0.838	0.838
0.88	0.876	0.877	0.878	0.879
0.92	0.917	0.918	0.919	0.919
0.96	0.959	0.959	0.959	0.96
1	1	1	1	1

**Figure 11:**
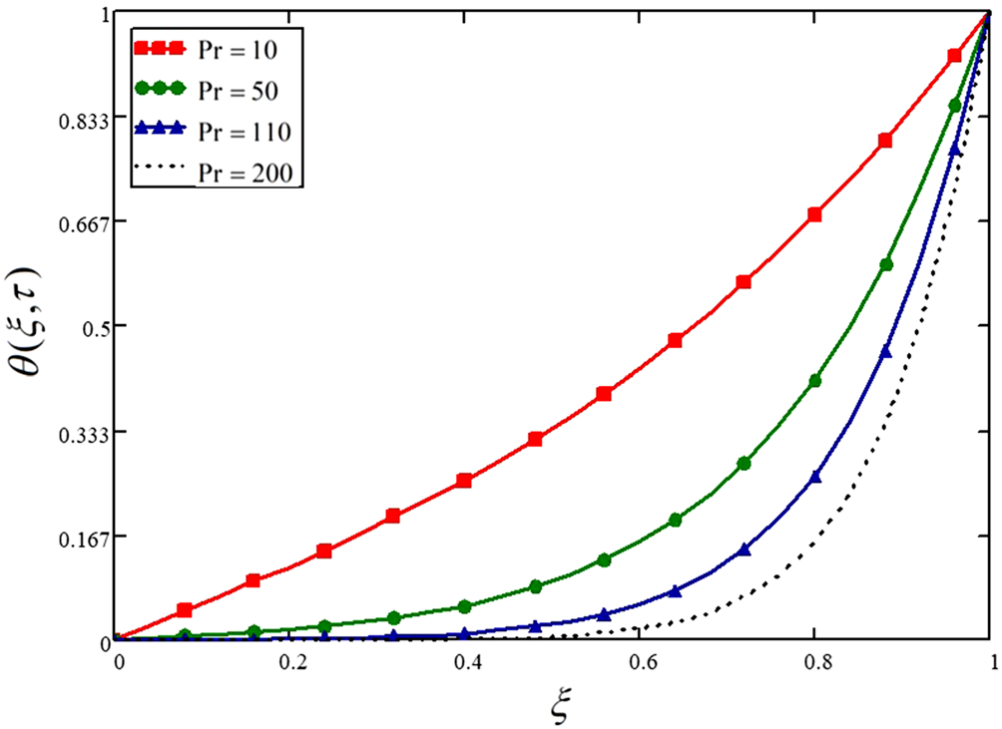
Influence of Prandtl number on the nanofluid velocity.

The influence of the Prandtl number on the temperature profile is presented in Fig. 11 and Table 11. Like the velocity profile, the temperature profile is also showing a decreasing trend for higher values of the Prandtl number, which is due to the weaker thermal forces.

**Table 11:** Variations in temperature profile against $\xi$ if $f(\tau ) = 1$ for different values of $\Pr$_._

$\xi$	$\theta (\xi ,\tau )$ at $\Pr = 10$	$\theta (\xi ,\tau )$ at $\Pr = 50$	$\theta (\xi ,\tau )$ at $\Pr = 110$	$\theta (\xi ,\tau )$ at $\Pr = 200$
0	0	0	0	0
0.04	0.023	2.657e^−3^	2.006e^−4^	8.104e^−6^
0.08	0.046	5.452e^−3^	4.305e^−4^	1.885e^−5^
0.12	0.069	8.529e^−3^	7.229e^−4^	3.564e^−5^
0.16	0.093	0.012	1.119e^−3^	6.364e^−4^
0.2	0.117	0.016	1.671e^−3^	1.112e^−4^
0.24	0.142	0.021	2.453e^−3^	1.919e^−4^
0.28	0.168	0.027	3.563e^−3^	3.283e^−4^
0.32	0.195	0.034	5.136e^−3^	5.57e^−4^
0.36	0.224	0.043	7.357e^−3^	9.374e^−4^
0.4	0.254	0.054	0.01	1.564e^−3^
0.44	0.285	0.067	0.015	2.589e^−3^
0.48	0.318	0.083	0.021	4.247e^−3^
0.52	0.354	0.103	0.029	6.904e^−3^
0.56	0.391	0.127	0.041	0.011
0.6	0.431	0.156	0.056	0.018
0.64	0.474	0.191	0.078	0.028
0.68	0.519	0.233	0.106	0.044
0.72	0.567	0.283	0.144	0.068
0.76	0.619	0.342	0.195	0.103
0.8	0.674	0.414	0.261	0.156
0.84	0.733	0.498	0.347	0.233
0.88	0.795	0.598	0.458	0.342
0.92	0.86	0.715	0.601	0.498
0.96	0.929	0.849	0.782	0.715
1	1	1	1	1

## Conclusion

In this study, a new approach is used to develop the fractional model of Casson nanofluid. Generalized Fourier’s law is used to fractionalize the model. A modern transformation is used to solve the model by the Laplace and Fourier transformation techniques. The obtained solutions are plotted and presented in tables. The primary outcomes of the present study are:

1. The new transformation is more reliable for the solution of the fractional model. It is easier to solve the fractional model using this transformation.
2. This transformation is reducing the computational time for finding the exact solutions to such problems and makes it easy to show that the solutions are satisfying the boundary conditions.
3. The velocity of the Casson fluid is higher for the greater values of $\beta$, which shows that the fluid will behave like a Newtonian viscous fluid for higher values of $\beta$.
4. The variations in all the profiles are shown for different values of $\alpha$. It is important here to mention that we have different lines for one value of time. This effect is showing the memory effect in the fluid, which cannot be demonstrated from the integer-order model.
5. The higher values of volume fraction of the nanoparticles making the engine oil more viscous, which may improve the lubrication of the oil.
6. The velocity of fluid is reducing for greater values of the Hartman number and is increasing for higher values of the Darcey parameter.

## List of symbols

$$\mu$$: Dynamic viscosity
$e_{ij}$: The $\left( {i,\,j} \right){\text{th}}$ component of deformation rate
$p_{y}$: The yield stress of non-Newtonian fluid
$\pi = e_{ij} e_{ij}$: The product of the component of deformation rate itself
$\pi_{c}$: The critical value of this product based on the non-Newtonian model
$\mu_{\gamma }$: The plastic dynamic viscosity
$u$: The fluid velocity in the $x$-direction
$$\Theta$$: The temperature
$\rho$: The fluid density
$\gamma_{C}$: The material parameter of Casson fluid
$\beta_{\Theta }$: The thermal expansion coefficient
$g$: The acceleration due to gravity
$c_{p}$: The specific heat capacity of fluids
$k$: The thermal conductivity
